# [^18^F]-HX4 PET/CT hypoxia in patients with squamous cell carcinoma of the head and neck treated with chemoradiotherapy: Prognostic results from two prospective trials

**DOI:** 10.1016/j.ctro.2020.04.004

**Published:** 2020-04-18

**Authors:** Sebastian Sanduleanu, Olga Hamming-Vrieze, Frederik W.R. Wesseling, Aniek J.G. Even, Frank J. Hoebers, Ann Hoeben, Wouter V. Vogel, Margot E.T. Tesselaar, Daniel Parvin, Harry Bartelink, Philippe Lambin

**Affiliations:** a The D-Lab, Dpt of Precision Medicine, GROW – School for Oncology, Maastricht University, The Netherlands; b Department of Radiation Oncology (Netherlands Cancer Institute – Antoni van Leeuwenhoek), Plesmanlaan 121, 1066CX Amsterdam, The Netherlands; c Department of Radiation Oncology (MAASTRO), GROW – School for Oncology and Developmental Biology, Maastricht University Medical Centre+, Doctor Tanslaan 12, 6229 ET Maastricht, The Netherlands; d Department of Medical Oncology, Maastricht University Medical Centre+, P.Debyelaan 25, 6229 HX Maastricht, The Netherlands; e Dpt. of Radiology and Nuclear Medicine, GROW – School for Oncology, Maastricht University Medical Centre+, The Netherlands; f Department of Nuclear Medicine ((Netherlands Cancer Institute – Antoni van Leeuwenhoek), Plesmanlaan 121, 1066CX Amsterdam, The Netherlands; g Department of Medical Oncology ((Netherlands Cancer Institute – Antoni van Leeuwenhoek), Plesmanlaan 121, 1066CX Amsterdam, The Netherlands; h MIM Software Inc., 25800 Science Park Drive – Suite 180, OH 44122, United States

**Keywords:** Head and neck, Radiaton therapy, Hypoxia, Positron emission tomography, [18F]-HX4, Prognosis

## Abstract

**Introduction:**

The presence of hypoxia in head-and-neck squamous cell carcinoma is a negative prognostic factor. PET imaging with [18F] HX4 can be used to visualize hypoxia, but it is currently unknown how this correlates with prognosis. We investigated the prognostic value of [18F] HX4 PET imaging in patients treated with definitive radio(chemo)therapy (RTx).

**Materials and methods:**

We analyzed 34 patients included in two prospective clinical trials (NCT01347281, NCT01504815). Static [18F] HX4 PET-CT images were collected, both pre-treatment (median 4 days before start RTx, range 1–16), as well as during RTx (median 13 days after start RTx, range 3–17 days). Static uptake at both time points (n = 33 pretreatment, n = 28 during RTx) and measured changes in hypoxic fraction (HF) and hypoxic volume (HV) (n = 27 with 2 time points) were analyzed. Univariate cox analyses were done for local progression free survival (PFS) and overall survival (OS) at both timepoints. Change in uptake was analyzed by comparing outcome with Kaplan-Meier curves and log-rank test between patients with increased and decreased/stable hypoxia, similarly between patients with and without residual hypoxia (rHV = ratio week 2/baseline HV with cutoff 0.2). Voxelwise Spearman correlation coefficients were calculated between normalized [18F] HX4 PET uptake at baseline and week 2.

**Results:**

Analyses of static images showed no prognostic value for [18F] HX4 uptake. Analysis of dynamic changes showed that both OS and local PFS were significantly shorter (log-rank P < 0.05) in patients with an increase in HV during RTx and OS was significantly shorter in patients with rHV, with no correlation to HPV-status. The voxel-based correlation to evaluate spatial distribution yielded a median Spearman correlation coefficient of 0.45 (range 0.11–0.65).

**Conclusion:**

The change of [18F] HX4 uptake measured on [18F] HX4 PET early during treatment can be considered for implementation in predictive models. With these models patients with a worse prognosis can be selected for treatment intensification.

## Highlights

• Early changes in hypoxia could be a promising biomarker to identify patients with unfavorable prognosis.
• Large differences were found between baseline and week 2 hypoxia PET signal.
• Neither baseline nor week 2 static parameters were associated with worse OS and local PFS.

## 1 Introduction

Overall 5-year survival rate of patients with head and neck squamous cell carcinomas (HNSCC) ranges from 40 to 65% [1], [2], [3]. Several prognostic factors have been recognized such as tumor stage at time of presentation and human papillomavirus (HPV) association [1], [4], [5], [6]. Cells in hypoxic areas may cause tumors to become resistant to radiotherapy and chemotherapy, increase tumor aggressiveness, angiogenesis and metastatic potential [7], [8], [9], [10], [11].

In recent years, the possibility of tailoring (pre-)treatment to biological tissue parameters such as hypoxia has emerged [12], [13]. This strategy employs biological tissue parameters to guide treatment intensity, for instance by selection of patients in need for treatment adaptation or by using biological tissue parameters for volume delineation of radio-resistant tumor parts [11].

Multiple PET tracers have been developed to non-invasively detect hypoxia, such as nitroimidazoles. The first generation nitroimidazole tracer ^18^F-Misonidazole (FMISO) has a slow clearance of unbound tracer resulting in a relative low tumor to background signal. Alternative tracers were developed such as the 2-nitroimidazole nucleoside analog [18F] HX4 [14]. This tracer has a high water solubility and fast clearance from non-hypoxic tissue, therefore generating a tracer with preferred pharmacokinetic properties [15]. Furthermore one phase I trial showed that [18F] HX4 PET imaging for the detection of hypoxia is not associated with any toxicity at any injection dose [16].

In a simulation study comparing 3 hypoxia tracers, [18F] HX4 showed the highest clearance and image contrast and the lowest background signal, followed by 18F-fluoroazomycin arabinoside (FAZA) and FMISO [17]. Furthermore, a high spatial reproducibility was observed by voxel-to-voxel comparisons and DICE similarity between repeated [18F] HX4 PET scans [18].

The prognostic potential of FAZA and FMISO PET has been previously described [19]. To the best of our knowledge there has not been any prognostic HNSCC study identifying hypoxic patients with [18F] HX4 PET.

The aim of this analyses was to investigate the prognostic value of [18F] HX4 imaging at baseline or at two weeks during radiotherapy treatment, as well as the prognostic value of the change in uptake between these time points.

## 2 Materials and methods

### 2.1 Patient selection and treatment

We analyzed 34 patients with stage II-IVA HNSCC included in two prospective clinical trials (NCT01347281 and NCT01504815) who underwent at least one [18F] HX4 PET scan. Both trials were approved by the Medical Ethics Review Committee and all patients gave written informed consent. Trial NCT01347281 was a diagnostic trial with the aim to: (i) determine if tumor hypoxia can be accurately visualized with [18F] HX4 PET imaging in head and neck tumors, (ii) correlate the [18F] HX4 PET images with blood and tissue markers, (iii) investigate the quality and optimal timing of [18F] HX4 PET imaging and (iv) compare [18F] HX4 PET uptake with [18F] FDG PET uptake before and after treatment. Trial NCT01504815 was designed as a randomized interventional trial with the aim to: (i) evaluate tumor dose redistribution impact on loco-regional control and toxicity by comparing 70 Gy standard dose distribution to adaptive inhomogeneous dose distribution ranging from 64 to 84 Gy (mean 74 Gy) to primary tumor depending on FDG-PET uptake, (ii) develop treatment specific tumor response predictors for patient tailored treatment, including [18F] HX4 PET imaging before and during treatment.

For this analysis, institutional review board approval was obtained. All 34 patients had histological or cytological confirmed HNSSC of the oral cavity (n = 1), oropharynx (n = 17), hypopharynx (n = 6) or larynx (n = 10), T1-4, any N, non-metastatic tumors (Table 1). All curative radiotherapy (RTx) schedules were allowed. 17 patients received a standard radiation dose of 70 Gy in 35 fractions in 47 days, 3 patients included in the experimental arm of the NCT01504815 trial received a mean dose of 74 Gy in 35 fractions in 47 days and 14 patient received an accelerated schedule to 68 Gy consisting of 34 fractions of which the final 10 fractions were given twice a day to secure an overall treatment time of 38 days. The majority of patients received concurrent cisplatin or cetuximab (Table 1, supplementary material Appendix A).

**Table 1:** Baseline patient characteristics. A detailed description of the trials, patient inclusion, stage and tumor subsite is presented in Appendix A.

Characteristics	Entire cohort (n = 34)	
	Median (range)	

Age	60 (44–77)	
GTV_prim_ (cm^3^)	14.52 (2.05–75.54)	

	Number of pts	

WHO PS		
0	11	(32)
1	22	(65)
2	1	(3)

Clinical TNM (T)		
cT1	1	(3)
cT2	5	(15)
cT3	16	(47)
cT4	12	(35)

Clinical nodal stage (N)		
cN0	14	(41)
cN1	4	(12)
cN2a	1	(3)
cN2b	11	(32)
cN2c	4	(12)
cN3	0	(0)

HPV status (P16)		
Positive	11	(32)
Negative	6	(18)
Unknown	17	(50)

RTx dose (Gy) primary/N+/elective		
68/68/52.7	14	(41)
70/70/54.25	17	(50)
74/70/54.25	3	(9)

Treatment type		
Concurrent cisplatin-radiation	20	(59)
Radiotherapy only, accelerated	8	(23)
Cetuximab-radiation, accelerated	6	(18)

Tumor site		
Oropharynx	17	(50)
Larynx	10	(29)
Hypopharynx	6	(18)
Oral cavity	1	(3)

*Abbreviations:* GTV_prim_, primary gross tumor volume; WHO PS, World Health Organization Performance Status; HPV, human papilloma virus; p16, tumor suppressor gene encoded by the CDKN2A gene.

### 2.2 Image acquisition

All patients underwent a pre-treatment planning CT (pCT) with a personalized immobilization mask. [18F] HX4 PET-CT images were acquired pre-treatment (median 4 days before start RTx, range 1–16) as well as during RTx (median 13 days after start RTx, range 3–17 days) using high-resolution full-ring PET/CT scanners (Philips Gemini 16 and Siemens Biograph 40 scanner). Static PET images of the head and neck area in the same immobilization mask were acquired 4 h after intravenous administration of an average (±SD) dose of 427 ± 55 MBq [18F] HX4 in the NCT01347281 trial and 386 ± 25 MBq [18F] HX4 in the NCT01504815 trial.

The images were reconstructed using scanner-specific parameters in accordance with each facility’s standard procedure, including at least attenuation and scatter correction.

The 4 h post-injection (p.i.) time point is related to a plateau phase in tracer uptake associated with optimal imaging properties [15]. More details regarding the acquisition parameters/protocol and scanner types are presented in Appendix A.

### 2.3 Image evaluation of [18F] HX4

Gross tumor volumes for primary tumor (GTVprim) were manually defined on the pCT by experienced radiation oncologists in both participating centers, using a standard head and neck window and level, and considering clinical information and related MR images when available.

For both datasets the clinical GTV_prim_ delineations defined on the pCTs were transferred to the baseline and week 2 [18F] HX4 PET image associated low dose CT (HX4 CT) by means of rigid registration with Mirada software (Mirada Medical, Oxford, UK). Air and bone were filtered out and the delineations were manually adjusted where needed.

The uptake of [18F] HX4 was evaluated in the GTV_prim_ volume after the [18F] HX4 PET and CT dimensions were matched (taking into account differences in pixel coordinates, pixel spacing, pixel size) and the GTV_prim_ contour was projected onto the HX4 PET. The mean background uptake of [18F] HX4 in non-hypoxic normal tissue was measured in a spacious volume in both trapezoid muscles throughout 3 slice levels (SUV_muscle_). Each voxel on [18F] HX4 PET was classified as hypoxic or non-hypoxic based on various cutoffs (1.2, 1.4 and 1.6) for the tumor to background ratio (TBR), which was calculated as voxel SUV uptake/mean muscle uptake. The hypoxic fraction was calculated as the number of hypoxic voxels/total number of tumor voxels using Reggui software (OpenReggui version r1357, Louvain-la-Neuve, Belgium). The hypoxic volume was calculated as the number of hypoxic voxels * voxel size.

A hypoxic volume was defined if the volume exceeded >0.01 cm^3^, this is larger than the voxel size (Appendix A). A residual hypoxic volume (rHV) was defined as the ratio of the hypoxic volume in week 2 of RTx and at baseline with a cutoff of 0.2 [20] Voxel wise Spearman correlation coefficients were calculated with Mim software version 6.9.0 (Cleveland, Ohio, United States of America, www.mimsoftware.com) on the [18F] HX4 PET voxels within the GTVprim (propagated from the baseline onto the week 2 [18F] HX4 CT by volume) after initial rigid registration of the baseline and week 2 [18F] HX4 CTs.

### 2.4 Statistical analysis

The statistical analysis was performed using R studio software, version 3.3.4 (http://www.R-project.org). The R packages used in this study were stats, rms, survival and survminer.

Univariate cox regression analysis was performed to assess independent predictors for overall survival (OS) and local progression free survival (PFS). The following covariates were tested in the group of patients with baseline [18F] HX4 PET (n = 33), and also in the group of patients with a [18F] HX4 PET performed during treatment (n = 28): Age, WHO PS, T-stage, N-stage, tumor location, number of pack years, treatment type, hypoxic fraction (HF), hypoxic volume (HV), HPV-status (p16 staining), HF times HV and GTVprim volume. Due to the low sample size multivariable cox regression analysis was omitted.

Statistical significance levels were two-sided, reported with a significance level of 0.05, however, to account for multiple testing, adjusted P-values through the Benjamini-Hochberg procedure were also reported.

In the group of patients with two [18F] HX4 PET scans the difference in outcome between patients with HV/HF increase compared to no increase (HF or HV stable or decreasing between baseline and week 2) was estimated by Kaplan-Meier curves and by a log-rank test. Independent samples t-tests were used for normally distributed continuous data and Fisher exact tests for categorical variables.

## 3 Results

A baseline [18F] HX4 PET was available in 33 patients, a [18F] HX4 PET during treatment was available in 28 patients and evaluation of the change of uptake signal could be done in 27 patients with scans available at both time points.

Median follow up in the whole group of 34 patients was 26.0 months. Local progression free survival at 2 and 5 years was 73.5% and 64.7% respectively, while overall survival at 2 and 5 years was 76.5% and 67.6% respectively.

### 3.1 Static baseline analyses

[18F] HX4 PET hypoxic volumes (cm^3^_)_ based on the GTV_prim_ varied notably among all 33 tumors assessed at baseline with a range of 0.0 to 27.69 (median: 2.09) cm^3^ according to a TBR cutoff of 1.4.

A hypoxic volume (>0.01 cm^3^) could be identified in 30/33 tumors according to TBR 1.2, in 26/33 tumors according to TBR 1.4 and in 21/33 tumors according to TBR 1.6 (Appendix B). In the remainder of the analyses, a TBR of 1.4 was used. In the TBR 1.4 group, 1/7 (14%) patients without a hypoxic volume were HPV positive, while in the patients with a hypoxic volume, 11/26 (42%) patients were HPV positive.

Cox regression analysis revealed no significant independent baseline predictors (after multiple testing adjustment) for local PFS or OS (Table 2, supplementary Appendix D). The Spearman’s correlation coefficient (ρ_S_) at baseline between baseline tumor volume and pre-treatment hypoxic volume was 0.77 (P < 0.001).

**Table 2:** Univariate cox regression in the prediction of local progression-free survival (LPFS).

Predictors	Baseline (n = 33)			Week 2 RTx (n = 28)		
	Beta	Hazard Ratio (95% CI)	P-value (adjusted)	Beta	Hazard Ratio (95% CI)	P-value (adjusted)
Age	−0.032	0.97 (0.9–1.0)	0.38 (0.71)	−0.02	0.98 (0.9–1.1)	0.65 (0.94)
WHO PS	0.99	2.7 (0.95–7.6)	0.062 (0.08)	0.89	2.4 (0.72–8.2)	0.15 (0.39)
T-stage	−0.37	0.69 (0.33–1.4)	0.31 (0.67)	0.29	1.3 (0.5–3.6)	0.56 (0.91)
N-stage	0.15	1.2 (0.63–2.2)	0.63 (0.91)	−0.056	0.95 (0.46–2.0)	0.88 (0.98)
Tumor location	0.42	1.5 (0.98–2.4)	0.06 (0.39)	0.44	1.6 (0.91–2.7)	0.11 (0.36)
Pack years	0.0013	1.0 (0.98–1.0)	0.91 (1.00)	0.0017	1.0 (0.98–1.0)	0.90 (0.98)
Treatment type	0.46	1.6 (0.81–3.1)	0.18 (0.59)	0.31	1.4 (0.58– 3.2)	0.47 (0.91)
Hypoxic fraction	−1.9	0.14 (0.0063–3.3)	0.23 (0.60)	0.95	2.6 (0.17–40.0)	0.50 (0.91)
Hypoxic volume	−0.00092	1.0 (0.9–1.1)	0.99 (1.00)	0.17	1.2 (1.0–1.3)	0.0092 (0.85)
HPV	−20.0	2.7 * 10^−9^ (0–inf)	1.00 (1.00)	−20	2.9 * 10^−9^ (0– inf)	1.00 (1.00)
(HF × HV)	−0.059	0.94 (0.75–1.2)	0.62 (0.91)	0.24	1.3 (0.96–1.7)	0.093 (0.36)
GTV_prim_ volume	0.027	1.0 (0.99–1.1)	0.17 (0.59)	0.055	1.1 (1.0–1.1)	0.013 (0.08)

### 3.2 Static week 2 analyses

Twenty eight patients had a second [18F] HX4 PET-CT scan. Analyses in this subgroup of the clinical parameters and static [18F] HX4 uptake in week 2 showed no significant predictors for OS and local PFS (after multiple testing adjustment) in univariate Cox analysis (Table 2, Appendix D).

The Spearman’s correlation coefficient (ρ_S_) at week 2 of RTx between tumor volume and hypoxic volume was 0.73 (P < 0.001).

### 3.3 Change between baseline and week 2

**Fig. 1:**
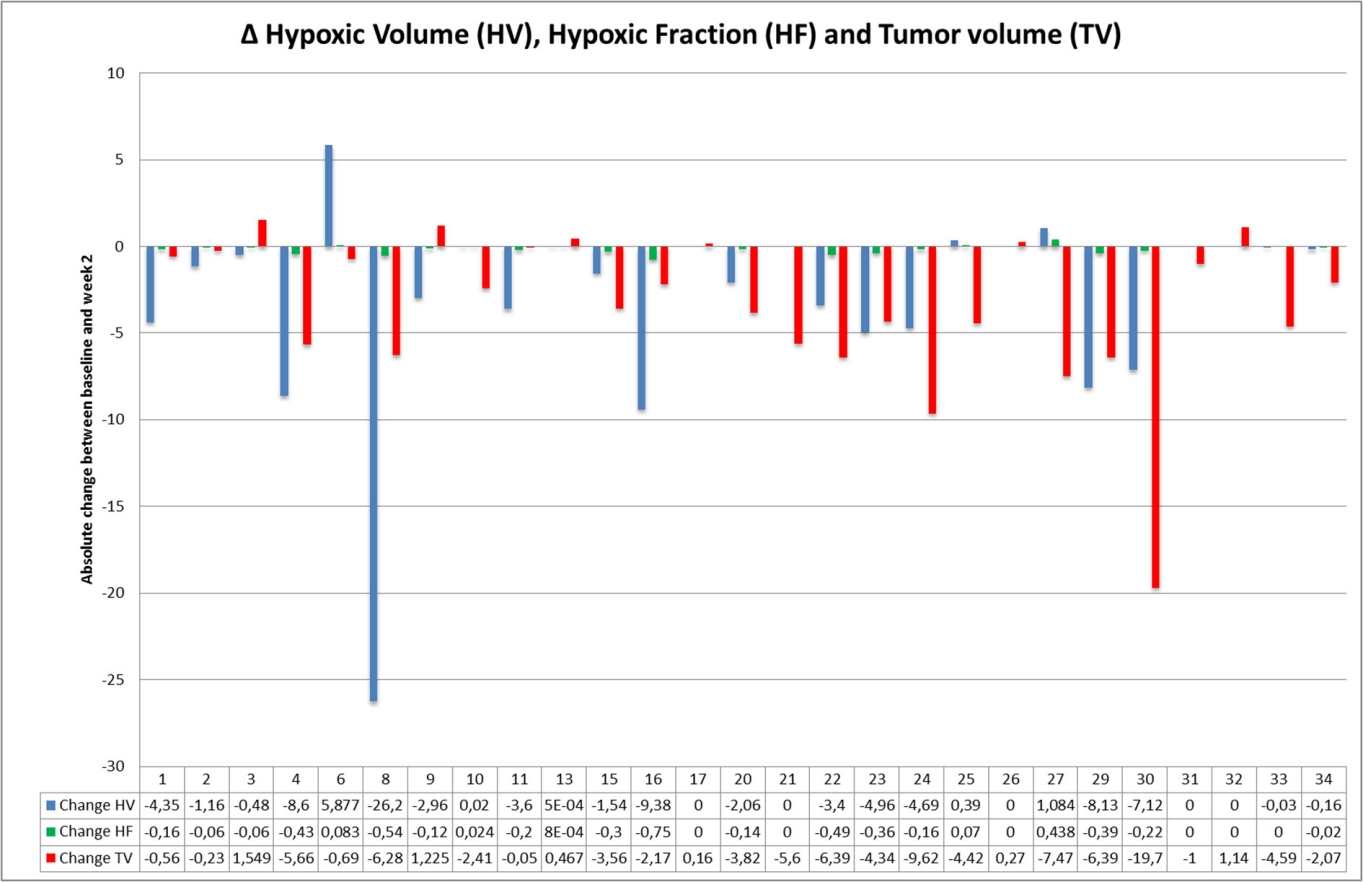
Absolute change (week 2 RTx – baseline) in hypoxic volume (cm3), hypoxic fraction (%) and tumor volume (cm3) according to TBR 1.4.

Analyses of dynamic change in the 27 patients with [18F] HX4 PET scans at both time points showed a large baseline-week 2 inter-patient change in GTVprim tumor volume and hypoxic volume/ fraction (Fig. 1). An HF and HV increase in week 2 was seen in the same 5/27 patients. Both corresponding OS and local PFS was significantly lower (log-rank P < 0.05) in the group of patients with an increase in hypoxic fraction/hypoxic volume (Fig. 2a, Fig. 2b). There was no significant difference (P > 0.05) between the HPV-status in the group of patients that increased in hypoxic fraction/hypoxic volume compared to those that were stable or had a decrease.

**Fig. 2a:**
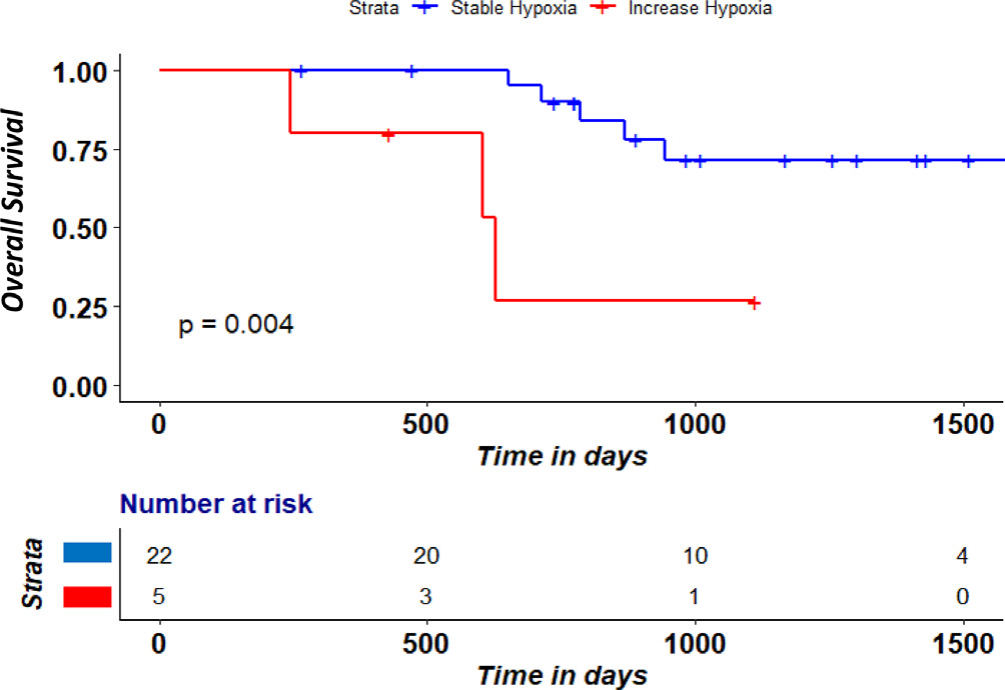
Overall survival according to increase in hypoxic fraction/hypoxic volume between pre-RT and after week 2 RTx.

**Fig. 2b:**
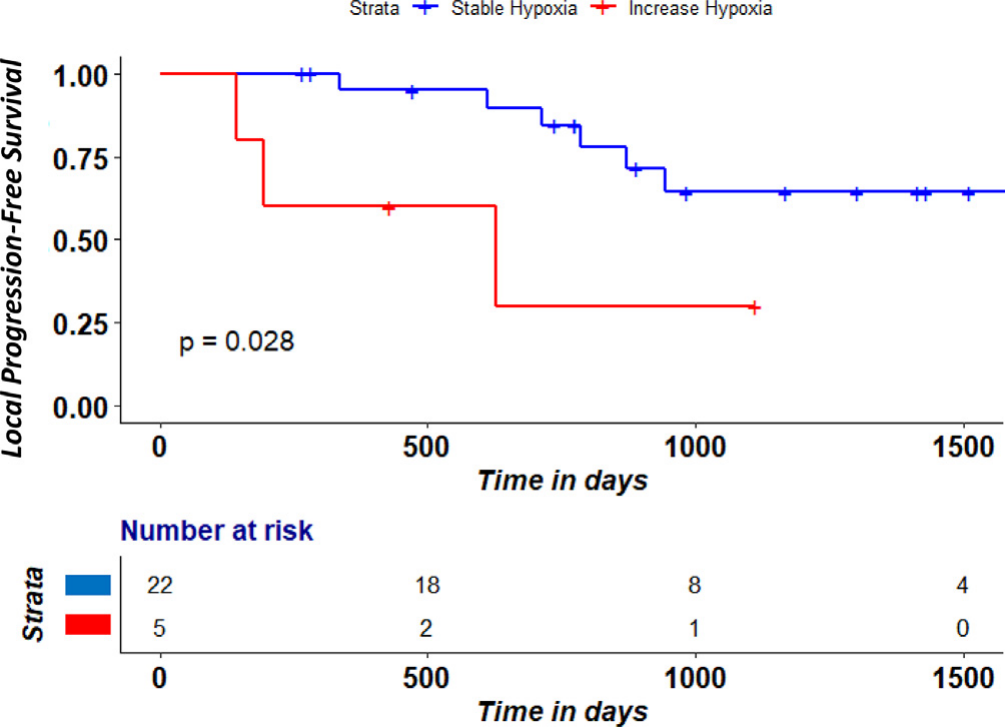
Local-Progression-free survival according to increase in hypoxic fraction/ hypoxic volume between pre-RT and after week 2 RTx.

A residual hypoxic volume (rHV) was identified in 6/27 patients (rHV defined as > 0.2). Overall survival was significantly lower (P = 0.02) in the group with rHV (Fig. 2c), Local PFS was not (P = 0.12). Nevertheless, in the residual hypoxia group the median local PFS was 18.3 months compared to 25.9 months in the group without rHV (Fig. 2d). No significantly different patient characteristics between rHV groups were found (Table 3).

**Fig. 2c:**
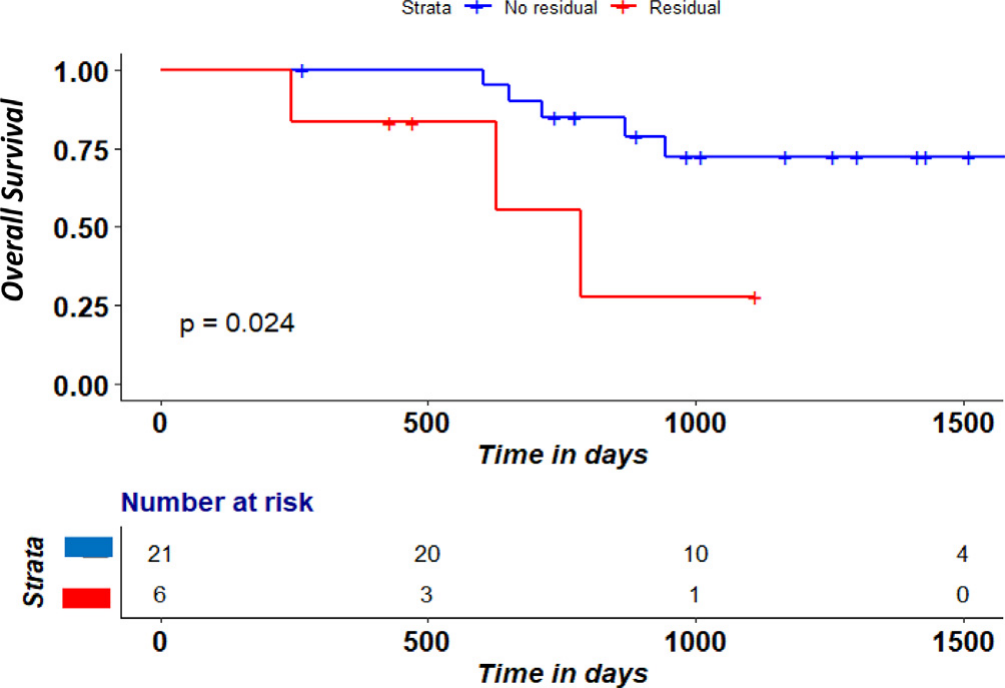
Overall survival according to rHV ratio between week 2 RTx and pre-RTx according to rHV cutoff of 0.2.

**Fig. 2d:**
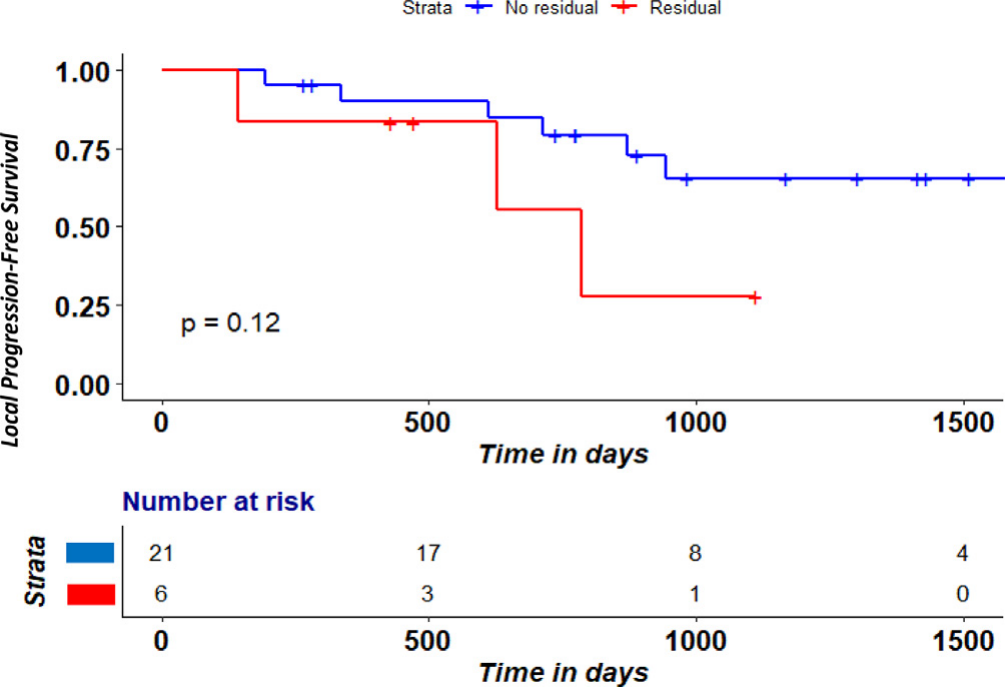
Local-Progression-free survival according to rHV ratio between week 2 RTx and pre-RTx according to rHV cutoff of 0.2.

**Table 3:** Comparison of patient characteristics between decrease/ stable hypoxia group and increase in hypoxia group.

Baseline – week 2 RTX Δ Hypoxia for TBR 1.4 (n = 27)						
Patient characteristics	Decrease/stable hypoxia group (n = 22)	Hypoxia increase group (n = 5)	P-value	rHV ≤ 0.2 group (n = 21)	rHV > 0.2 group (n = 6)	P-value
Median Δ GTV_prim_ – CT (cm^3^, (range))	−2.86 (−19.67–1.55)	−2.41 (−7.47–0.47)	0.976^a^	−3.56 (−19.68–1.14)	−1.55 (−7.47–1.55)	0.441^a^

Median age (years, (range))	60 (44–77)	57 (50–72)	0.744^a^	60 (44–77)	64.5 (56–72)	0.303^a^

WHO PS						
0	7	2	1.000^b^	5	4	0.174^b^
1	14	3		15	2	
2	1	0		1	0	

HPV status (n, (%))						
P16 positive	8	1	0.539^b^	7	2	0.607^b^
P16 negative	3	2		3	2	
Unknown	11	2		11	2	

TNM (T)						
1	0	0	0.826^b^	0	0	0.700^b^
2	4	0		4	0	
3	10	3		10	3	
4	8	2		7	3	

Nodal stage (N)						
0	11	2	0.805^b^	12	1	0.063^b^
1	3	0		3	0	
2	8	3		6	5	

RTx dose (Gy)						
68	11	1	0.320^b^	12	1	0.144^b^
70	10	3		8	4	
74	1	1		1	1	

Treatment						
Accelerated radiotherapy	5	1	1.000^b^	6	0	0.274^b^
Concurrent cisplatin-radiation	14	4		12	6	
Concurrent accelerated Cetuximab-radiation	3	0		3	0	

Tumor site						
Oropharynx	10	3	0.861^b^	9	4	0.108^b^
Larynx	8	1		9	0	
Hypopharynx	3	1		2	2	
Oral cavity	1	0		1	0	

*Abbreviations*: GTV, gross tumor volume; CT, computed tomography; WHO PS, World Health Organization Performance Status; HPV, human papilloma virus; p16, tumor suppressor gene encoded by the CDKN2A gene.

a The p-values were obtained by independent samples *t*-test or Wilcoxon rank sum test.

b The p-value were obtained by the Fisher-exact test.

The voxel-based correlation analysis yielded a median Spearman’s correlation coefficient (ρ_Spearman_) of 0.45 between [18F] HX4_pre_ and [18F] HX4_w2_ (range 0.11–0.65) (supplementary Appendix C).

## 4 Discussion

In this pooled analysis of two prospective clinical trials we showed that change in hypoxia early during treatment measured with [18F] HX4 PET is a promising biomarker to identify patients with an unfavorable prognosis. An increase of hypoxic fraction/hypoxic volume and the presence of residual hypoxia in week 2 were associated with a significantly worse prognosis. Neither baseline nor week 2 static parameters were associated with worse OS and local PFS.

Recently, literature discussing early response prediction during treatment has been emerging. Early effects of radiotherapy can lead to changes in tumoral permeability and increased blood flow that may result in opening of previously non-perfused vessels and neo-angiogenesis [17]. Indeed, studies reporting on hypoxia response measurement with PET show that a rapid decrease of hypoxia in both primary GTV as well as positive lymph nodes in the early weeks of treatment is mostly an independent prognostic factor [18], [19]. Whether re-oxygenation occurs might reflect underlying radio-sensitivity of the tissue and therefore predict outcome. Lock et al. [20] evaluated re-oxygenation during the course of treatment with FMISO-PET in an exploratory cohort and a validation cohort, both consisting of 25 patients. Instead of a fixed cutoff value, the pre-treatment FMISO-PET of each individual patient served as intra-patient control to calculate the residual hypoxia volume at week 1, 2 and 5. A significant decrease in loco-regional control for tumors with residual hypoxia could be shown in the exploratory cohort and was successfully validated in the validation cohort. The strongest predictive value was found in the second week of treatment [20]. Mortensen et al. [21] used FAZA PET at baseline in a cohort of 40 patients with HNSCC to show a significant improved disease free survival in non-hypoxic tumors compared to hypoxic tumors. Only 13 patients had a second FAZA PET during treatment after a median of 14 days of which most had no residual hypoxic volume. Treatment failure occurred in 4/6 patients with residual hypoxic volume compared to 2/7 patients with no residual hypoxia. Our results on residual HX4-measured hypoxia are in line with both Mortensen and Lock [20], [21].

It is unclear how the predictive value of hypoxia imaging relates to other functional imaging methods e.g. other PET tracers (i.e. metabolism or proliferation), functional MRI (dynamic contrast enhanced or diffusion weighted), CT-perfusion or CT derived radiomics. Most likely, in the future, multi-factorial prediction models will be developed, combining clinical, pathological and imaging information to determine individual tumor responsiveness for personalized therapy.

In the quest to enhance the therapeutic ratio, dose escalation is considered in patients with adverse prognostic factors. The question which volume to use for dose escalation is not yet solved, be it either the entire primary tumor or a radioresistant sub-volume. Dose escalation studies for both approaches are in progress or planned to be performed [12], [20]. Toxicity of dose escalation is related to treatment volume and the gain in tumor control could be larger with a focal dose escalation to a smaller radioresistant volume to allow a higher maximum tolerated dose with equal toxicity [22], [23], thereby making a biological target to guide dose escalation attractive. However, whether hypoxia imaging is accurately reproducible is uncertain. Conflicting literature reports about hypoxic PET signal reproducibility are provided [13], [24], [25], [26], [27]. Zegers et al. analyzed the correlation of [18F] HX4 uptake with a voxel-wise analyses on scans 2 days apart [25]. Most patients showed a moderate to good correlation. In our cohort, the correlation coefficient between the location of the HV at baseline and at week 2 PET was low, on average 0.45. Several reasons for a lack of consistency can be acknowledged. Hypoxia is a dynamic process with both a chronic and acute component depending on the degree and dynamics of perfusion. Delineation methods of hypoxic volumes bare uncertainty, as well as defining a tumor to background cutoff value for which consensus does not exist. In this current study a TBR threshold of 1.4 was used based upon a previous [18F] HX4 PET imaging study in head and neck cancer patients [25]. Furthermore, this method is sensitive to placement of the background region of interest and signal noise within the background. Uncertainty increases with the decrease in contrast between tumor and background [28]. On top, the PET voxel size is relatively large and represents a spatial average of the hypoxia signal in the corresponding tissue, thus diluting the PET signal (partial volume effect).

Another approach to enhance treatment outcome is to modify hypoxia. In some clinical trials hypoxia-activated prodrugs (HAP’s) have failed to demonstrate efficacy in terms of overall survival, presumably due to the lack of patient selection eligible for hypoxia modification (e.g. highly hypoxic tumors, specific tumor phenotype) [29], [30], [31]. In contrast, recently a preclinical study has underlined the promising efficacy of evofosfamide in aggressive HPV-negative HNSCC with regard to time to starting tumor volume after radiotherapy [32]. In the past, clinical trials have shown that benefits of targeting hypoxia in head and neck cancer patients are mainly seen in HPV negative cancer patients [33], [34], [35]. The biologic mechanisms that underlie this phenomenon are not well understood, though it is believed that this is not related to the frequencies of hypoxic tumors among HPV-positive and HPV-negative tumors but more to inherent radiosensitivity [36]. Based on our results, it seems logical to also stratify patients in HAP-trials according to early hypoxia response, besides more common used factors such as HPV and volume. A relatively high association between hypoxic volume and primary tumor volume was found in our analysis, which corresponds to findings in both preclinical/clinical studies in primary solid malignancies [37], [38]. One should be aware that this does not necessarily hold true for e.g. micrometastatic tumors, as these have been shown to exhibit high levels of hypoxia [37].

Some limitations of this study include: (i) Pooling of imaging data between two hospitals might lead to inconsistencies in the calculation of the hypoxic fractions and hypoxic volumes. (ii) Small sample size, with the result that e.g. our cox-models could not be validated with multivariate analyses. (iii) Heterogeneity between the two populations of patients with regard to treatment. (iv) Differences in [18F] HX4 scan time periods prior to treatment as well as for week 2 might influence PET results due to increase/decrease in (acute) hypoxia. (v) Unbalanced group sizes in KM-analysis.

In conclusion, the change of [18F] HX4 uptake measured with PET early during treatment can be considered as a prognostic factor. With these models patients with a worse prognosis can be selected for treatment intensification or hypoxia targeting, although the [18F] HX4 signal in itself seems less appropriate due to spatial instability to use for focal target definition.

## Conflict of interest disclosure

No actual or potential conflicts of interest exist. Dr. Philippe Lambin reports, within and outside the submitted work, grants/sponsored research agreements from Varian medical, Oncoradiomics, ptTheragnostic, Health Innovation Ventures and DualTpharma. He received an advisor/presenter fee and/or reimbursement of travel costs/external grant writing fee and/or in kind manpower contribution from Oncoradiomics, BHV, Merck and Convert pharmaceuticals. Dr Lambin has shares in the company Oncoradiomics SA and Convert pharmaceuticals SA and is co-inventor of two issued patents with royalties on radiomics (PCT/NL2014/050248, PCT/NL2014/050728) licensed to Oncoradiomics and one issue patent on mtDNA (PCT/EP2014/059089) licensed to ptTheragnostic/DNAmito, three non-patentable invention (softwares) licensed to ptTheragnostic/DNAmito, Oncoradiomics and Health Innovation Ventures. Dr. Bartelink and Dr. Hamming-Vrieze report grants from EU 7th framework program (ARTFORCE – n° 257144), during the conduct of the study. Dr. Sebastian Sanduleanu reports a grant from NWO (Nederlandse Organisatie voor Wetenschappelijk Onderzoek), during the conduct of the study.
