# Supplementary material for: [18F]-HX4 PET/CT hypoxia in patients with squamous cell carcinoma of the head and neck treated with chemoradiotherapy: Prognostic results from two prospective trials
Source: Clin Transl Radiat Oncol. 2020 Apr 18;23:9–15. doi: 10.1016/j.ctro.2020.04.004 (PMC7184102; doi:10.1016/j.ctro.2020.04.004)
Supplement: Supplementary Data 1 [file mmc1.docx]

**Appendix A: Trial and HX4 PET/CT image parameters**

| Institute | MAASTRO | NKI-AVL |
| --- | --- | --- |
| Trial number | NCT01347281 | NCT01504815 |
| Number of patients | 22 | 12 |
| Main purpose | Diagnostic | Interventional |
| Treatment | Radiotherapy +/- concurrent chemotherapy: Radiotherapy to 68-70 Gy with concurrent Cetuximab, Cisplatin or nothing depending on disease status and patient condition according to hospital guidelines. | *Standard arm*: Cisplatin + conventional RT: Cisplatin 100mg/m2 on days 1, 22 and 43, with conventional RT 70 Gy. *Experimental arm*: Cisplatin + adaptive dose redistributed RT: Cisplatin, 100 mg/m2 on days 1, 22 and 43 with RT on primary tumor 64-84 Gy (mean 74 Gy) depending on FDG-PET uptake |
| PET/CT scanner | Biograph 40 PET/CT scanner (Siemens Healthcare, Erlangen, Germany) | Gemini TF 16 (Philips Healthcare, Best, The Netherland) |
| Patient position | Single position | Single position |
| Acquisition time | 4h post injection | 4h post injection |
| Slice thickness (mm) | 3.0 | 2.0 |
| Pixel spacing (mm) | 1.17x1.17 | 1.17x1.17 |
| Voxel size (cm^3^) | 0.0041 | 0.0027 |
| HX4 PET | HX4 PET before treatment and after 20 Gy +/- 4 Gy | HX4 PET before treatment and after 20 Gy +/- 4 Gy |

**Appendix B: Effect of TBR threshold on baseline hypoxic HV**


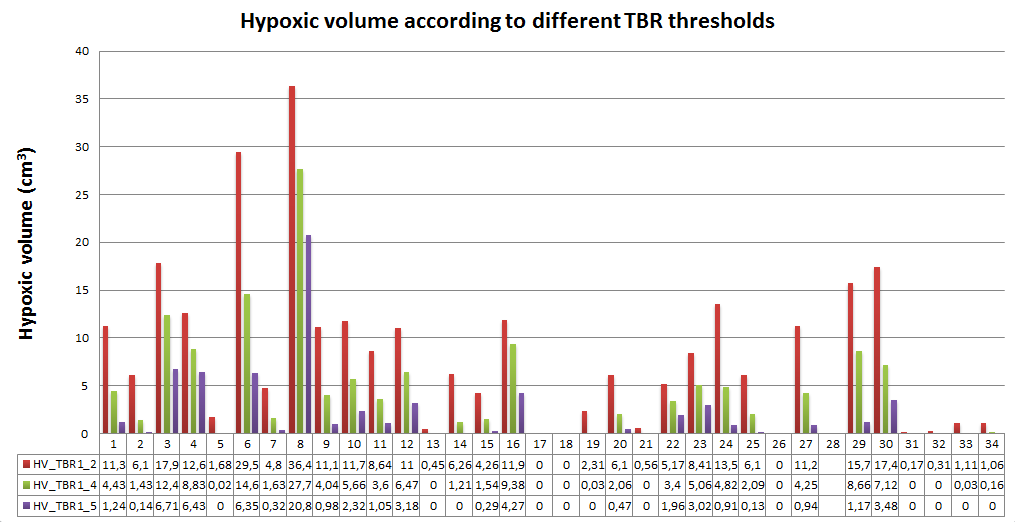


**Figure 1.** Baseline hypoxic volumes of thirty-four tumors according to different TBR cutoffs. The hypoxic volume was defined by HX4 PET/CT imaging as all voxels within GTV_HX4-T_ expressing a tumor-to muscle ratio above 1.2, 1.4 and 1.6 respectively.

**Appendix C: Voxel-wise correlation coefficients (ρS)**


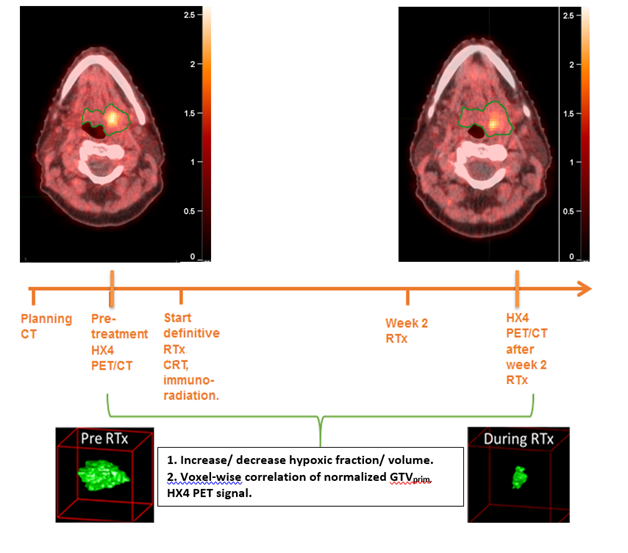


**Figure 2.** Voxelwise Spearman correlation coefficient (between baseline and week 2 RTx, within GTV_prim_).

**Appendix D: Univariate Cox regression for Overall Survival (OS)**

|  | **Baseline (n=33)** | | | **Week 2 RTx (n=28)** | | |
| --- | --- | --- | --- | --- | --- | --- |
| **Predictors** | **Beta** | **Hazard Ratio (95% CI)** | **P-value (adjusted)** | **Beta** | **Hazard Ratio (95% CI)** | **P-value (adjusted)** |
| Age | -0.061 | 0.94 (0.87 - 1.0) | 0.11 (0.54) | -0.06 | 0.94 (0.86 -1.0) | 0.21 (0.48) |
| WHO PS | 0.95 | 2.6 (0.82 - 8.1) | 0.10 (0.54) | 0.85 | 2.3 (0.60 – 9.1) | 0.22 (0.48) |
| T-stage | -0.42 | 0.66 (0.31 - 1.4) | 0.29 (0.54) | 0.29 | 1.3 (0.48 - 3.7) | 0.59 (0.85) |
| N-stage | 0.30 | 1.3 (0.70 - 2.6) | 0.30 (0.54) | 0.019 | 1 (0.48 – 2.2) | 0.96 (1.00) |
| Tumor location | 0.31 | 1.4 (0.87 - 2.2) | 0.17 (0.54) | 0.39 | 1.5 (0.84 - 2.6) | 0.17 (0.48) |
| Pack years | -0.0019 | 1 (0.97 - 1.0) | 0.88 (1.00) | -0.00088 | 1 (0.97 – 1.0) | 0.95 (1.00) |
| Treatment type | 0.41 | 1.5 (0.73 - 3.1) | 0.27 (0.54) | 0.18 | 1.2 (0.46 – 3.1) | 0.71 (0.92) |
| Hypoxic fraction | -1.8 | 0.17 (0.0061 - 4.6) | 0.29 (0.54) | 1.3 | 3.8 (0.25 - 58) | 0.34 (0.63) |
| Hypoxic volume | 0.0013 | 1 (0.9 - 1.1) | 0.98 (1.00) | 0.19 | 1.2 (1.1 -1.4) | 0.0044 (0.06) |
| HPV | -20 | 3.0 * 10^-9^ (0-Inf) | 1 (1.00) | -20 | 3.2* 10^-9^ (0-Inf) | 1.00 (1.00) |
| (HF x HV) | -0.04 | 0.96 (0.76 - 1.2) | 0.73(1.00) | 0.29 | 1.3 (1.0 - 1.8) | 0.049 (0.21) |
| GTV_prim_ volume | 0.02 | 1 (0.98 - 1.1) | 0.33 (0.54) | 0.053 | 1.1 (1.0 - 1.1) | 0.024 (0.16) |
